# Evaluating the Impact of Extended Kalman Filter Odometry on the Performance of 2D LiDAR SLAM Algorithms

**DOI:** 10.3390/s26175468

**Published:** 2026-08-29

**Authors:** Christian Merrick, Vidya K. Nandikolla

**Affiliations:** Department of Mechanical Engineering, California State University Northridge, Northridge, CA 91330, USA; christian.merrick.633@my.csun.edu

**Keywords:** autonomous mobile robots, simultaneous localization and mapping (SLAM), sensor fusion, Extended Kalman Filter (EKF), LiDAR, localization, Robot Operating System (ROS)

## Abstract

Accurate localization and mapping are essential for autonomous mobile robots operating in unknown environments. This study investigates the impact of Extended Kalman Filter (EKF)-based sensor fusion on the performance of three widely used two-dimensional (2D) LiDAR Simultaneous Localization and Mapping (SLAM) algorithms: GMapping, Karto SLAM, and SLAM Toolbox. Wheel encoder longitudinal velocity and inertial measurement unit (IMU) yaw angular velocity were fused using an EKF and compared with raw wheel odometry using the MIT Stata Center dataset. Localization performance was evaluated both before and after SLAM using translational and rotational Absolute Pose Error (APE) across multiple trajectory segments. Five repeated executions were performed for each SLAM configuration to characterize run-to-run variability. Prior to SLAM, EKF-filtered odometry reduced translational APE root mean square error (RMSE) by approximately 61–75% and rotational APE RMSE by approximately 65–77% relative to raw odometry. After SLAM, translational differences between the two odometry sources were substantially smaller and varied according to the evaluated algorithm and trajectory, while rotational performance exhibited larger and less consistent changes. These results demonstrate that substantial improvements in upstream odometry accuracy do not necessarily produce proportional improvements in final SLAM localization and that the influence of sensor fusion varied across the evaluated SLAM algorithm and trajectory segments, providing practical guidance for selecting localization strategies in autonomous mobile robots.

## 1. Introduction

Autonomous navigation is a fundamental capability of intelligent mobile robots, enabling them to operate with minimal or no human intervention in complex and dynamic environments. Reliable navigation requires a robot to perceive its surroundings, estimate its position, build a representation of the environment, and generate safe trajectories while avoiding obstacles. These capabilities are essential for a wide range of applications, including warehouse automation, industrial inspection, autonomous vehicles, precision agriculture, search and rescue, planetary exploration, and environmental monitoring.

Recent advances in sensing technologies and computing have significantly improved the performance of autonomous navigation systems. Modern mobile robots commonly integrate multiple sensors, including wheel encoders, inertial measurement units (IMUs), two-dimensional (2D) LiDAR, RGB-D cameras, ultrasonic sensors, and Global Navigation Satellite Systems (GNSS). Because each sensor has its own strengths and limitations, sensor fusion techniques are widely used to combine complementary measurements and provide more accurate and reliable estimates of the robot’s motion [1,2].

One of the key challenges in autonomous navigation is simultaneous localization and mapping (SLAM), where a robot must estimate its pose while building a map of an unknown environment. Over the past two decades, numerous 2D LiDAR-based SLAM algorithms have been developed, including GMapping, Karto SLAM, Hector SLAM, Cartographer, and SLAM Toolbox. These algorithms differ in their approaches to scan matching, pose estimation, loop closure, and map optimization, resulting in different levels of localization accuracy, computational cost, and robustness [3]. Consequently, the performance of a SLAM algorithm depends not only on the environment, but also on the quality of the localization information available to it.

For many LiDAR-based SLAM algorithms, wheel encoder odometry provides the initial estimate of robot motion between consecutive laser scans. Although wheel odometry is inexpensive and easy to implement, its accuracy gradually degrades because of wheel slip, uneven terrain, encoder quantization, and accumulated integration errors. These errors can reduce localization accuracy and degrade the quality of the generated map. To address this problem, wheel encoder measurements are often fused with IMU data using an Extended Kalman Filter (EKF), which combines measurements from multiple sensors while accounting for their uncertainties to produce a more reliable estimate of the robot pose [4,5].

Previous studies have compared the localization and mapping performance of multiple 2D LiDAR SLAM algorithms under different operating conditions [5,6,7,8]. However, relatively few studies have specifically investigated how improvements in the upstream odometry accuracy propagate through different SLAM algorithms when evaluated under otherwise consistent experimental conditions.

This paper investigates the effect of EKF-based odometry on the performance of three widely used 2D LiDAR SLAM algorithms: GMapping, Karto SLAM, and SLAM Toolbox. The SLAM algorithm, LiDAR input, playback procedure, and evaluation method were kept constant while comparing two localization pipelines: raw wheel odometry and EKF-filtered odometry. Localization accuracy was assessed using the Absolute Pose Error (APE) root mean square error (RMSE).

The main contribution of this work is an evaluation of how improvements in upstream odometry accuracy propagate through different 2D LiDAR SLAM algorithms to influence final localization performance. This study quantifies localization error both before and after SLAM using raw wheel odometry and EKF-filtered odometry. The results provide practical insight into the importance of odometry quality for LiDAR-based SLAM and offer guidance for selecting appropriate localization strategies in autonomous mobile robot applications.

The remainder of this paper is organized as follows. Section 2 reviews the background on LiDAR-based SLAM, EKF-based sensor fusion, and the trajectory evaluation metrics used in this study. Section 3 describes the dataset, experimental methodology, and implementation of the evaluated SLAM algorithms. Section 4 presents the experimental results and compares localization performance. Section 5 discusses the findings, and Section 6 concludes the paper and outlines future work.

## 2. Background

### 2.1. Simultaneous Localization and Mapping (SLAM)

Simultaneous Localization and Mapping (SLAM) is one of the fundamental problems in mobile robotics. It enables a robot to construct a map of an unknown environment while simultaneously estimating its position within that map. These two tasks are closely coupled: accurate mapping depends on reliable localization, while accurate localization requires an accurate representation of the surrounding environment.

Most 2D LiDAR-based SLAM algorithms use a localization source, such as wheel encoder odometry, to provide an initial estimate of the robot’s motion between consecutive laser scans. This initial pose estimate is then refined through scan matching, which aligns successive LiDAR scans to improve localization accuracy [9]. In graph-based SLAM methods, scan-matching results and other sensor observations are represented as constraints between robot poses in a graph. Pose graph optimization is then used to minimize accumulated localization errors and improve map consistency.

As the robot revisits previously explored areas, loop closure introduces additional constraints that reduce accumulated drift and improve the global consistency of both the trajectory and the generated map. Although LiDAR-based SLAM algorithms follow this general framework, they differ in how odometry, scan matching, loop closure, and graph optimization are implemented [3]. As a result, their localization and mapping performance can vary depending on the quality of the odometry used as the initial motion estimate.

### 2.2. EKF-Based Sensor Fusion

Wheel encoder odometry provides a simple and computationally efficient estimate of robot motion and is widely used in mobile robotics. However, its accuracy gradually degrades because of wheel slip, uneven terrain, encoder quantization, and accumulated integration errors. These errors can significantly affect the localization accuracy of SLAM algorithms, particularly during long trajectories.

To improve odometry accuracy, wheel encoder measurements are commonly fused with data from an inertial measurement unit (IMU) using an Extended Kalman Filter (EKF) [4,10]. The EKF recursively estimates the robot’s state by combining the motion prediction obtained from wheel odometry with measurements from the IMU while accounting for the uncertainty associated with each sensor.

During the prediction step, the robot state is propagated using the system motion model. During the correction step, IMU measurements are incorporated to update the predicted state and reduce accumulated errors. The resulting EKF-filtered odometry can provide a more reliable estimate of the robot’s position and orientation than wheel encoder odometry alone, making it a suitable localization input for LiDAR-based SLAM algorithms.

### 2.3. Overview of Evaluated SLAM Algorithms

Three widely used 2D LiDAR-based SLAM algorithms were evaluated in this study: GMapping, Karto SLAM, and SLAM Toolbox.

GMapping is based on a Rao-Blackwellized particle filter that estimates the robot trajectory using multiple weighted particles while simultaneously constructing an occupancy grid map. It has become one of the most widely used SLAM algorithms because of its robustness and relatively low computational requirements [6,8,11].

Karto SLAM is a graph-based SLAM algorithm that represents robot poses as nodes connected through constraints derived from sensor observations. Pose graph optimization is then performed to minimize accumulated localization errors and improve global map consistency. Karto SLAM is well-known for producing accurate maps, particularly in environments where reliable loop closures are available [6,8,12].

SLAM Toolbox is a modern graph-based SLAM framework developed for the Robot Operating System (ROS) [13,14]. It extends traditional graph-based approaches by providing improved loop closure detection, pose graph optimization, lifelong mapping capabilities, and support for long-term autonomous operation. These features enable improved localization performance and greater flexibility in complex environments.

To investigate the influence of odometry quality, each SLAM algorithm was evaluated twice using the same recorded dataset. The first evaluation used raw wheel encoder odometry, while the second used EKF-filtered odometry generated by fusing wheel encoder and IMU measurements. By keeping the dataset, LiDAR input, SLAM algorithm configuration, playback procedure, and evaluation method constant, the effect of improved odometry on localization performance can be evaluated under consistent evaluation conditions.

### 2.4. Trajectory Evaluation Metrics

Evaluating localization accuracy is essential for assessing the performance of SLAM algorithms. By comparing the estimated robot trajectory with a corresponding ground truth trajectory, trajectory-based metrics can provide quantitative measures of how accurately a SLAM algorithm estimates the robot’s pose throughout navigation.

For planar mobile robots, each pose is an element of the two-dimensional Special Euclidean group, SE(2), which describes planar rigid-body motion using two translational coordinates (x, y) and one rotational coordinate (θ) [15] (p. 77). Since the robot operates on a planar surface, SE(2) provides a standard mathematical framework for representing rigid-body motion in two-dimensional environments, combining translation and rotation into a single pose representation.

One of the most widely used trajectory evaluation metrics is the Absolute Pose Error (APE), which measures the Euclidean distance between corresponding poses of the estimated and reference trajectories after trajectory alignment [16]. APE quantifies the accumulated localization error over an entire trajectory and is commonly summarized using the root mean square error (RMSE), mean error, and maximum error. Lower values indicate closer agreement between the estimated and reference trajectories, corresponding to higher localization accuracy.

## 3. Materials and Methods

**Figure 1:**
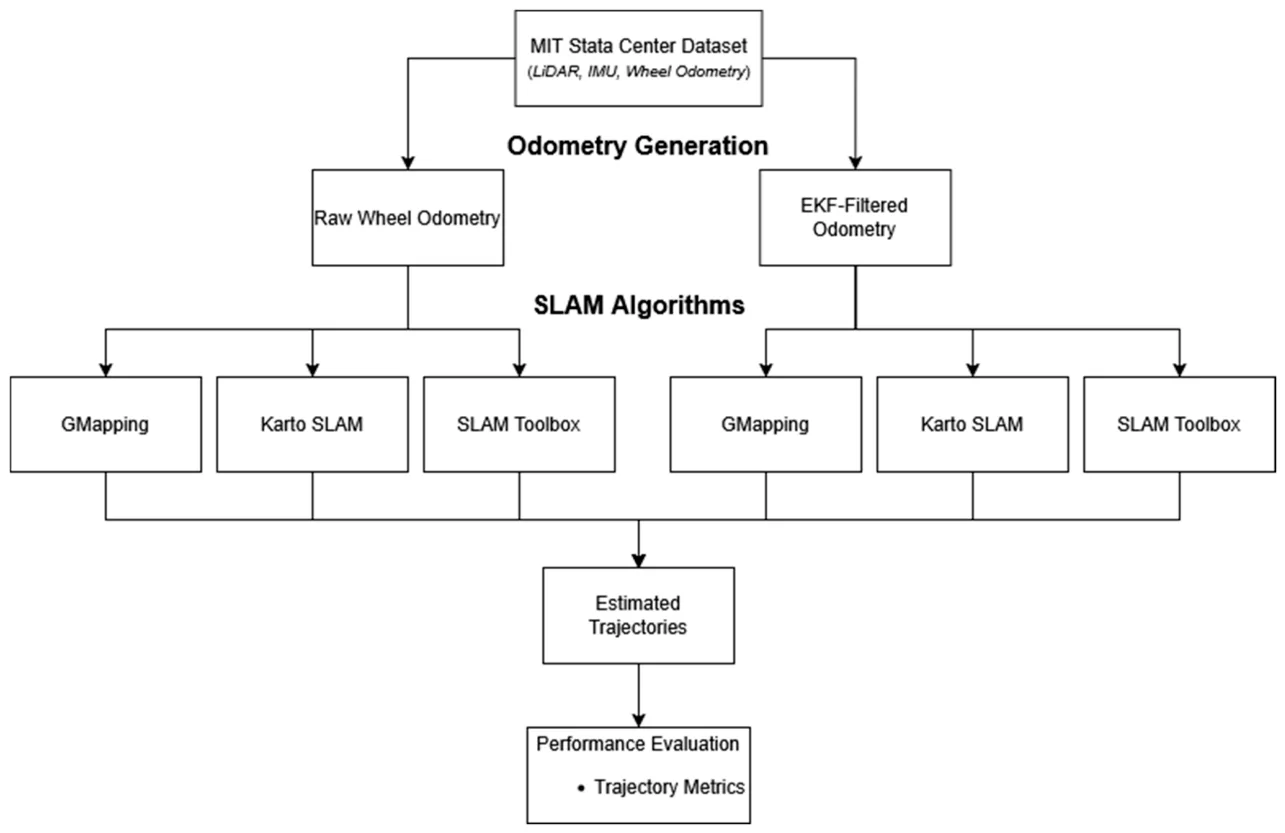
Experimental overview of the influence of odometry quality on SLAM performance.

The MIT Stata Center dataset, containing LiDAR, IMU, and wheel odometry measurements, was processed into two localization sources: raw wheel odometry and EKF-filtered odometry (Figure 1). Each localization source was independently provided as input into each of the evaluated SLAM algorithms.

### 3.1. Dataset

The experimental data used in this study were obtained from the Massachusetts Institute of Technology (MIT) Stata Center dataset, which was collected using the PR2 research robot (Willow Garage, Menlo Park, CA, USA). The dataset is publicly available in ROS bag format, enabling direct playback and processing within the Robot Operating System (ROS Melodic Morenia; Open Robotics, Mountain View, CA, USA). The dataset used in this work, 2012-04-06-11-15-29.bag, contains a complete traversal of the second floor of the MIT Stata Center and has been widely used to evaluate mobile robot localization and mapping algorithms [17].

The ROS bag contains synchronized data from multiple sensors, including a Hokuyo UTM-30LX 2D LiDAR (Hokuyo Automatic Co., Ltd., Osaka, Japan), wheel encoder odometry, and inertial measurements from a MicroStrain 3DM-GX2 IMU (MicroStrain, Inc., Williston, VT, USA). In addition, the dataset includes coordinate frame transformations, a ground-truth trajectory, and an EKF-based localization estimate.

### 3.2. Odometry Generation

To ensure complete control over the localization pipeline, only the raw wheel encoder odometry, IMU measurements, and their corresponding coordinate frame transforms were extracted from the original ROS bag. All other data streams were removed to reduce unnecessary computational overhead during playback. The EKF-filtered odometry provided with the dataset was intentionally excluded so that sensor fusion could be implemented and evaluated using a consistent methodology throughout this study.

During preliminary inspection of the dataset, the measurement covariance associated with the longitudinal wheel velocity in the incoming odometry message was found to be 4 × 10^−6^ (m/s)^2^, which was substantially smaller than the observed measurement uncertainty. Because this message-level covariance is used by robot_localization to weight the fused velocity measurement, an unrealistically small value can cause the EKF to place excessive confidence in the wheel encoder velocity measurements, reducing its ability to properly account for measurement uncertainty [10]. Therefore, the covariance associated with longitudinal wheel velocity was reassigned prior to EKF fusion with an empirically determined value. This was conducted by conducting residual analysis with reference to the time-synchronized ground-truth trajectory. This produced a velocity residual variance of 0.011317 (m/s)^2^, and so a new measurement covariance value of 0.01 (m/s)^2^ was implemented. Ground truth was only used for offline characterization of the wheel velocity measurement uncertainty and was not provided to the EKF as a measurement input or used to correct the EKF state during filtering. However, because this covariance calibration used the same reference trajectory later used for evaluation, the resulting EKF configuration is treated as ground-truth-informed, and this dependency is identified as a limitation of the study.

For the IMU input, only the yaw angular velocity, *ω_z_*, was selected for sensor fusion. Its measurement covariance was specified as 1.44 × 10^−4^ (rad/s)^2^ based on the specifications provided for the MicroStrain 3DM-GX2 IMU.

To investigate the effect of sensor fusion on SLAM performance, two sets of evaluation ROS bags were generated from the original dataset. The first used raw wheel encoder odometry as the localization source, while the second used EKF-filtered odometry obtained by fusing the wheel encoder and IMU measurements. The same dataset, LiDAR input, playback procedure, and evaluation method were used for both localization pipelines.

**Figure 2:**
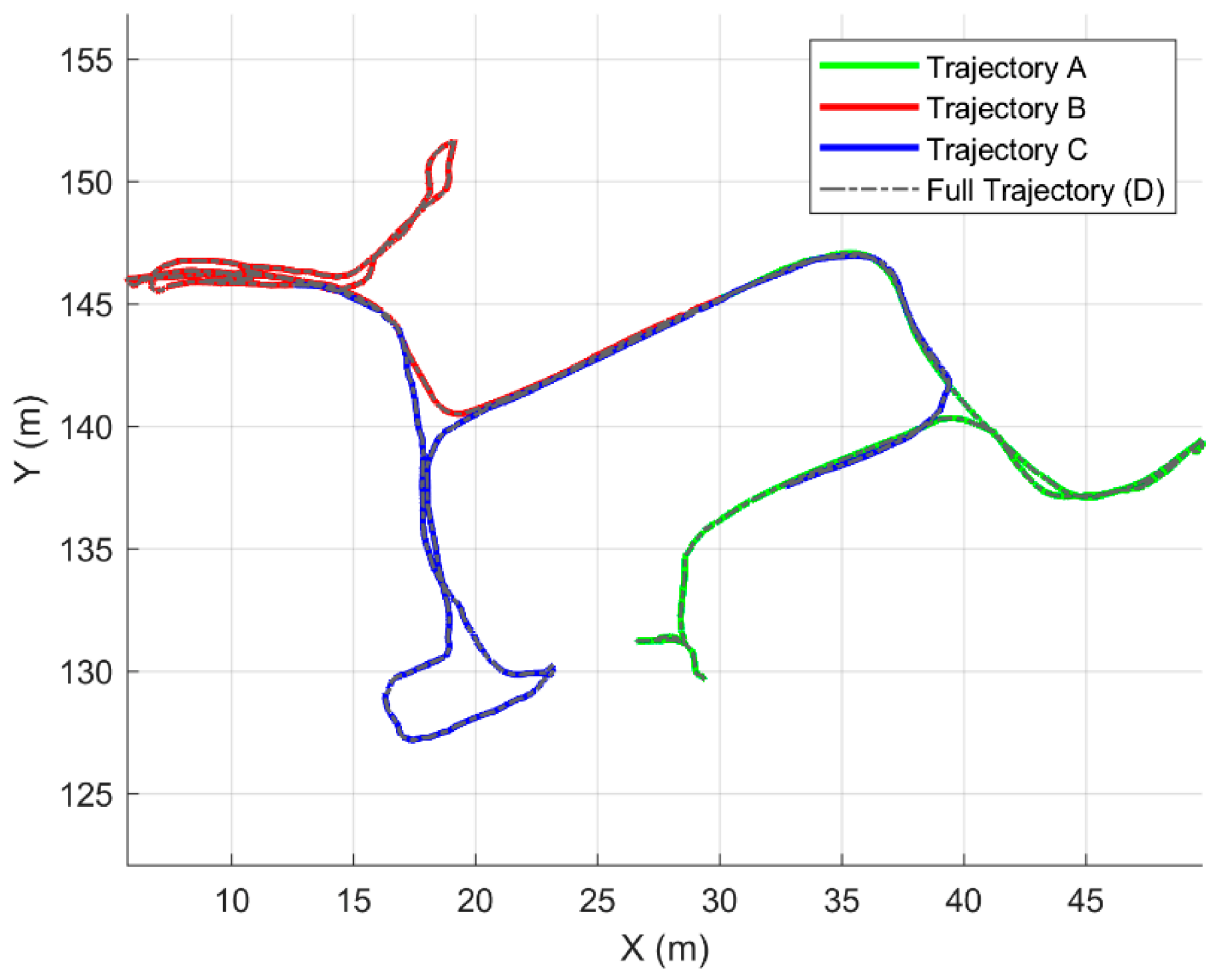
Ground-truth trajectory of the MIT Stata Center dataset showing the four evaluation trajectories. Trajectories A–C represent individual segments of the dataset, while Trajectory D corresponds to the complete route.

To evaluate localization performance over trajectories with different characteristics, the original dataset was divided into four evaluation trajectories. Trajectories A, B, and C represent individual sections of the complete route, whereas Trajectory D consists of the full dataset. Trajectories A–C were extracted from Trajectory D and therefore do not represent independent datasets. This segmentation enables the performance of each SLAM algorithm to be evaluated over both short and long trajectories while maintaining the same dataset, LiDAR input, playback procedure, SLAM configuration, and evaluation method across all evaluations. These trajectories can be seen in Figure 2.

#### 3.2.1. Raw Odometry

Raw wheel odometry estimates the robot pose by integrating wheel encoder measurements over time. In this study, it served as the baseline localization source and did not incorporate measurements from additional sensors such as the IMU. Although wheel odometry is computationally efficient and widely used in mobile robotics, its accuracy gradually degrades because of wheel slip, uneven terrain, encoder quantization, and accumulated integration errors. Nevertheless, it provides the initial motion estimate required by many LiDAR-based SLAM algorithms for scan matching.

ROS localization packages, such as robot_localization, publish an odom to base_link coordinate transform. This transform represents the robot’s position (*x*, *y*) and yaw angle (*ψ*) relative to its starting position and is used by SLAM algorithms as the initial pose for scan matching.

Because the raw odometry evaluation did not use robot_localization, the required odom to base_link was not available during playback. Therefore, a custom ROS node was created to broadcast the odom to base_link directly from the raw wheel odometry messages, enabling the SLAM algorithms to use the raw odometry as their localization input.

#### 3.2.2. EKF-Based Odometry

To evaluate the effect of sensor fusion on SLAM performance, wheel encoder odometry and IMU measurements were fused using an Extended Kalman Filter (EKF) implemented with the ROS robot_localization package (v2.6.12; Open Robotics, Mountain View, CA, USA). The EKF combines wheel encoder odometry with IMU measurements while accounting for the uncertainty of each sensor through the configured covariance matrices to estimate the robot’s pose.

The configured EKF fused forward wheel velocity *v_x_* and IMU yaw angular velocity *ω_z_*. The EKF output frequency was configured at 100 Hz to closely match the publication rate of the raw wheel odometry. The process noise covariance matrix and initial estimate covariance used by robot_localization are provided numerically in Table A1. The EKF was configured in two-dimensional mode with the odom frame used as the world frame.

### 3.3. SLAM Algorithm Procedure

Three 2D LiDAR-based SLAM algorithms were evaluated in this study using ROS 1 Melodic Morenia: GMapping v1.4.2, Karto SLAM (slam_karto) v0.8.1, and SLAM Toolbox (slam_toolbox) v1.1.6. Each algorithm was tested using the same LiDAR data, playback procedure, and evaluation method while comparing the two localization pipelines: raw wheel odometry and EKF-filtered odometry. This allowed the effect of odometry quality on localization to be evaluated under consistent evaluation conditions for each SLAM algorithm.

#### Algorithm Configuration

The selected SLAM algorithms were configured using their default parameter settings to provide a consistent basis for comparison. The only parameters modified were ones necessary to facilitate correct transform trees and data streams. Each algorithm received the same laser scan data while being evaluated with either the raw odometry or EKF-filtered odometry localization pipeline. Important parameters can be seen in Table 1.

### 3.4. Experimental Procedure

For each trajectory, the ROS bag was played back while the SLAM algorithm being evaluated was executed. The resulting data were recorded into separate evaluation ROS bags. This procedure was repeated five times for each combination of odometry source, trajectory, and SLAM algorithm, resulting in 120 SLAM executions. The estimated trajectories were then extracted from the ROS bags and converted into TUM trajectory format for quantitative evaluation using the evo Python package v1.37.0 [18].

Because the raw odometry and EKF-filtered odometry conditions originally used different mechanisms to publish the odom to base_link transform, an additional validation was performed to evaluate the effect of the TF publication mechanism. In the original EKF-filtered condition, the transform was published by robot_localization. For the validation, TF publication from the robot_localization node was disabled, and the EKF output topic /odometry/filtered was converted to TF using the same odometry-to-TF broadcaster used in the raw-odometry condition. This validation was performed for all three SLAM algorithms on Trajectories A-C with three repeated executions per condition. The resulting APE RMSE values were compared with the original EKF-filtered odometry results. The translational RMSE differences were small across all cases, with a maximum difference of 0.008 m, corresponding to 2.52%. Rotational RMSE differences were also small in magnitude, with a maximum difference of 0.099°. These results indicate that the TF publication mechanism did not materially affect the reported post-SLAM trajectory error trends. The full validation comparison is provided in Table A2.

### 3.5. Performance Evaluation

Localization performance was evaluated quantitatively by comparing each estimated trajectory to the corresponding ground-truth trajectory. Absolute Pose Error (APE) was used to quantify both translational and rotational differences between the estimated and ground-truth poses.

#### Trajectory Evaluation

The extracted estimated trajectories were aligned using the default *evo* package rigid-body SE(3) transformation prior to evaluation. Because the dataset is constrained to planar motion, vertical displacement, roll, and pitch are not estimated by these algorithms and the resulting alignment is restricted to translation in the x and y-directions along with a rotation about the z-axis while not changing the scale of the trajectory. This corresponds to an SE(2) alignment for the evaluated trajectories. This alignment removes differences in the initial pose so that the reported pose errors reflect localization performance rather than starting position offsets [16].

Three quantitative metrics were used for evaluation:

- Absolute Pose Error Root Mean Square Error (APE RMSE): The root mean square of the absolute pose differences between the poses along the estimated trajectory and ground truth trajectory. Also referred to as Absolute Trajectory Error.
- Absolute Pose Error Mean (APE Mean): The arithmetic mean of the absolute pose error between estimated and ground truth trajectory.
- Absolute Pose Error Max (APE Max): The maximum absolute pose error between the estimated and ground truth trajectory.

APE was calculated using the evo Python package [18]. A maximum timestamp difference of 0.03 s was enforced to ensure the proper alignment of ground-truth trajectory with estimated trajectory. This was necessary as the ground truth provided was published at 20 Hz. Translational APE was reported in meters, while rotational APE was reported in degrees. The relative change in APE RMSE was calculated such that positive values indicate reduced error with EKF-filtered odometry while negative values indicate increased error. An APE RMSE was calculated independently for each of the five repeated executions. The resulting values were summarized using the arithmetic mean and sample standard deviation for each algorithm, trajectory, and localization source.

## 4. Results

This section presents the quantitative evaluation of the selected SLAM algorithms using both raw wheel encoder odometry and Extended Kalman Filter (EKF)-filtered odometry. Localization performance was assessed by comparing the estimated trajectories with the ground truth using Absolute Pose Error (APE) metrics. A single dataset was analyzed and divided into three trajectory segments (Trajectories A–C), while Trajectory D represents the complete recorded trajectory.

### Localization Evaluation

To quantify the effect of EKF-based sensor fusion on localization accuracy, the trajectories generated using raw odometry and EKF-filtered odometry were compared with the corresponding ground truth trajectory. Table 2 summarizes the localization error metrics for each trajectory, including the APE root mean square error (RMSE), mean APE, and maximum APE.

**Figure 3:**
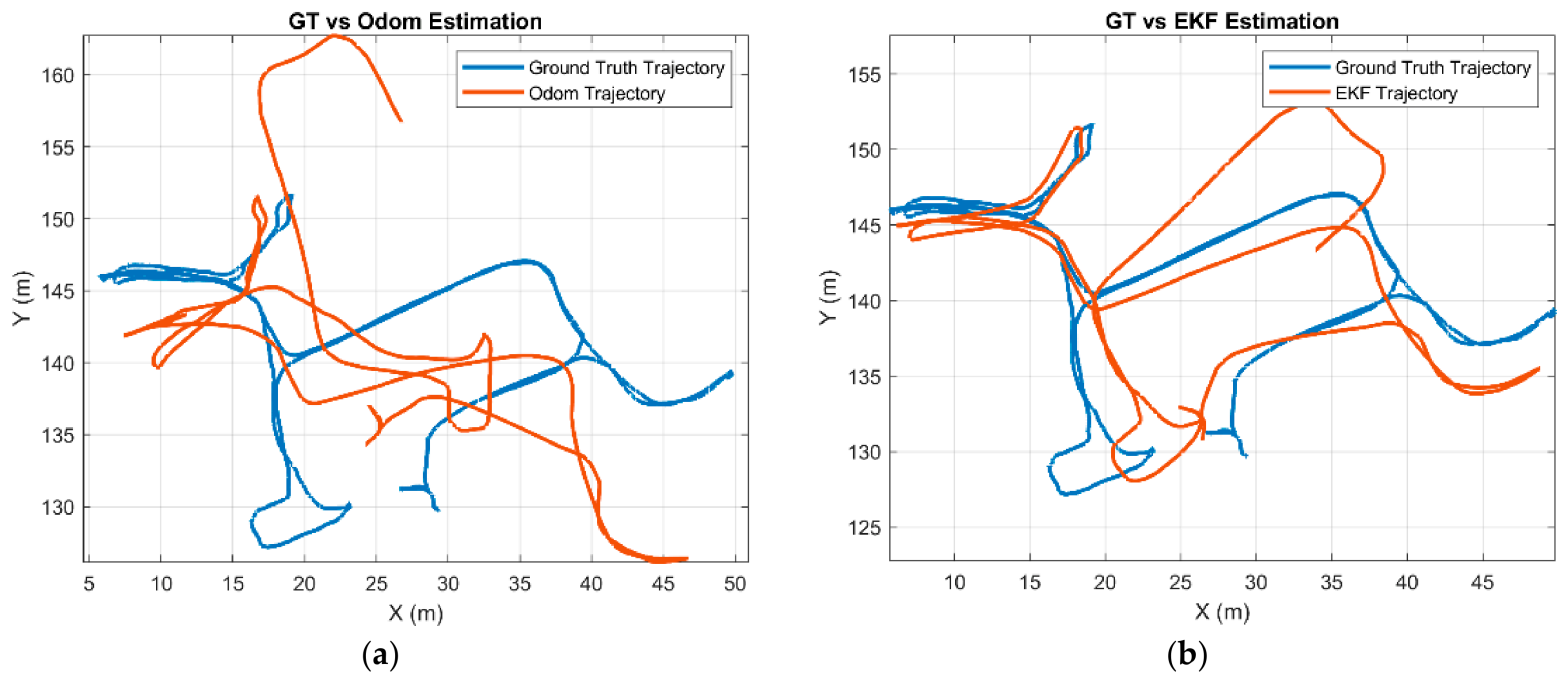
(**a**) Ground truth against raw odometry, Trajectory D; (**b**) ground truth against EKF-filtered odometry, Trajectory D.

Figure 3 compares the raw wheel odometry and EKF-filtered odometry trajectories against ground truth for the complete dataset (Trajectory D), while Table 2 summarizes the localization error metrics for each trajectory segment. The EKF trajectory follows the ground truth path more closely across all evaluated trajectories, reducing the overall trajectory error relative to raw odometry. This improvement is reflected in the APE metrics, where the EKF consistently produced lower RMSE, mean, and max APE values for every trajectory evaluated.

Across Trajectories A–D, the EKF reduced the translational APE RMSE by 60–75% and rotational APE RMSE by 65-77%, relative to the raw odometry. Similar reductions were observed for the mean and maximum APE values, indicating improved localization consistency throughout the trajectories. These results demonstrate that EKF-based sensor fusion substantially improved the accuracy of the odometry estimate prior to the SLAM refinement. The following section evaluates how these improvements influence the localization performance of the selected SLAM algorithms.

Despite the substantial pre-SLAM reductions in both translational and rotational errors, the post-SLAM results, as shown in Table 3, exhibited smaller and less consistent differences between the two odometry sources.

The statistical comparison shown in Table 4 indicates that several of the small translational percentage differences in Table 3 were not distinguishable from run-to-run variability. For example, the translational differences for GMapping Trajectories A, B, and D, and SLAM Toolbox A and D were not statistically distinguishable. In contrast, Karto SLAM Trajectories A–C showed statistically distinguishable increases in translational APE RMSE when using EKF-filtered odometry. Rotational APE RMSE exhibited more frequent statistically distinguishable differences, but the direction varied by algorithm and trajectory. These results support interpreting the post-SLAM effect of EKF-filtered odometry as algorithm and dataset dependent rather than uniformly beneficial.

**Figure 4:**
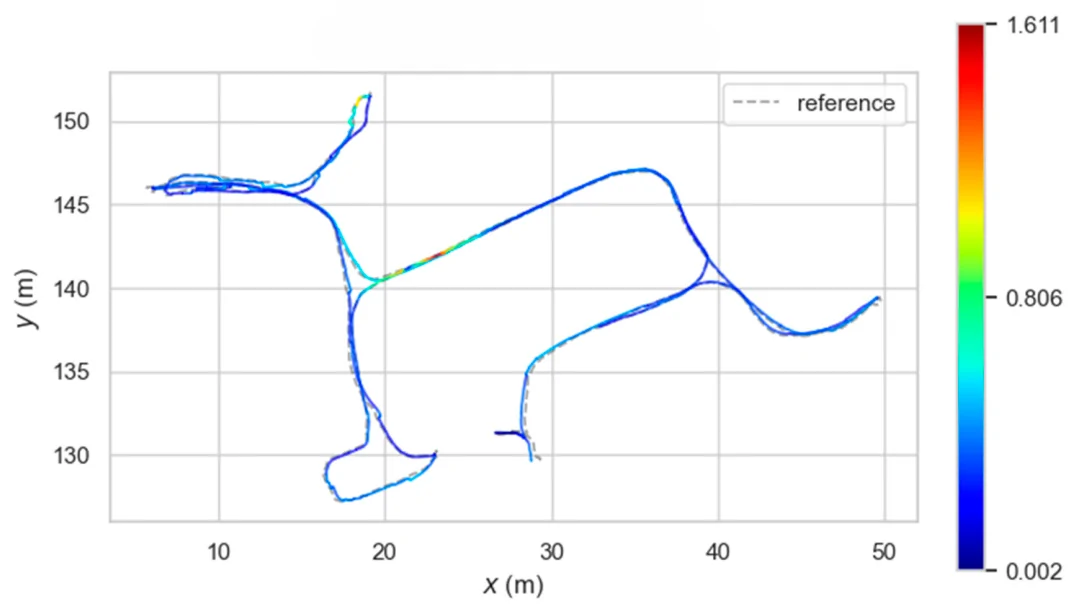
Estimated GMapping trajectory for Trajectory D using EKF-filtered odometry compared with the ground truth.

**Figure 5:**
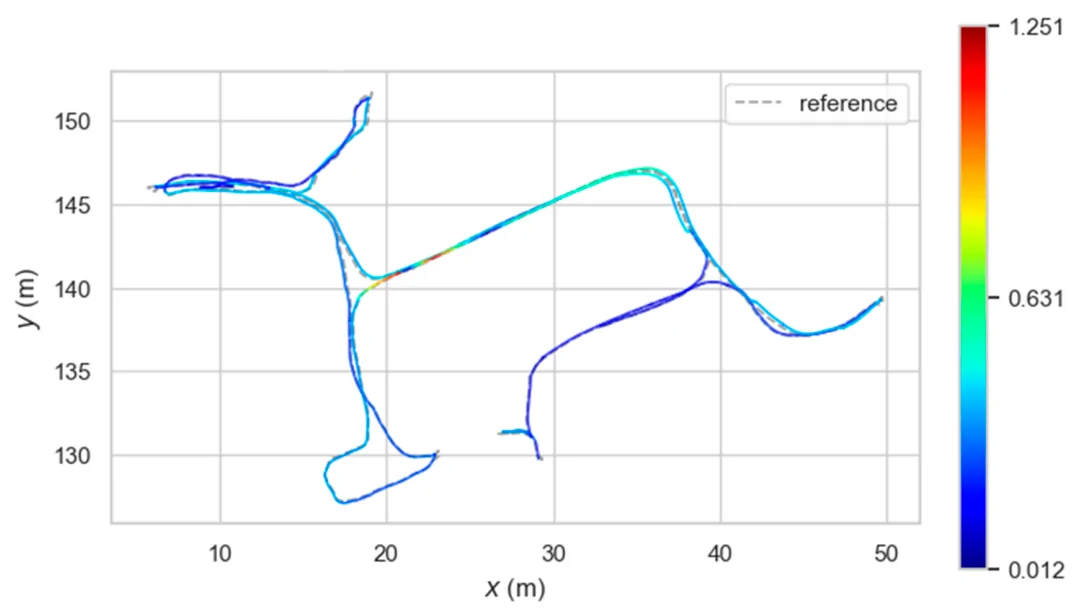
Estimated Karto SLAM trajectory for Trajectory D using EKF-filtered odometry compared with the ground truth.

**Figure 6:**
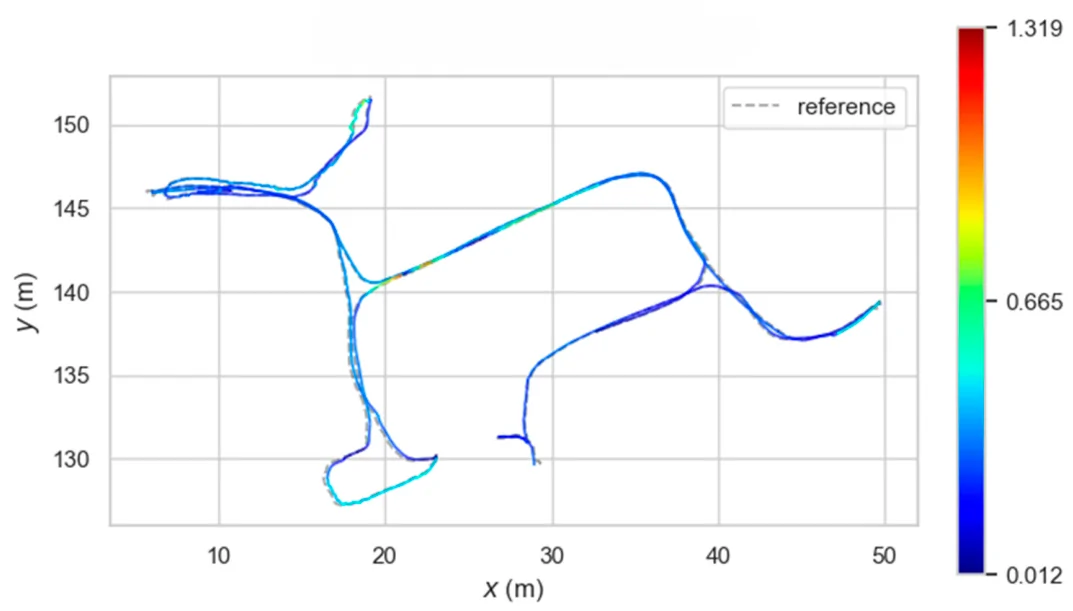
Estimated SLAM Toolbox trajectory for Trajectory D using EKF-filtered odometry compared with the ground truth.

Translational improvements were fairly minor compared to pre-SLAM improvements across all trajectories and algorithms and were not consistently favorable to either localization source. For GMapping, several EKF cases produced lower numerical errors, but the magnitude and statistical distinguishability varied by trajectory and metric. Karto SLAM and SLAM Toolbox exhibited the opposite trend, with EKF-filtering producing higher localization errors for a majority of the evaluated trajectories. Representative translational APE plots are shown for GMapping, Karto SLAM, and SLAM Toolbox in Figure 4, Figure 5 and Figure 6, respectively.

## 5. Discussion

### 5.1. Impact of Odometry Quality on SLAM Performance

The results show that improving odometry with an Extended Kalman Filter (EKF) had a measurable but relatively small effect on the overall localization performance of the evaluated 2D LiDAR SLAM algorithms. Before SLAM, EKF-based sensor fusion reduced the APE RMSE of the raw wheel odometry by approximately 60–75% translationally and 65–77% rotationally, demonstrating that combining wheel encoder and IMU measurements produced a substantially more accurate motion estimate. However, after SLAM execution, the change in APE RMSE was much less substantial and did not consistently favor the improved or raw localization source.

This indicates that odometry is only one part of the localization process. Although it provides the initial motion estimate, LiDAR scan matching provides an additional source of pose correction by aligning consecutive laser scans with the surrounding environment. The results suggest that much of the benefit gained from the improved odometry may be absorbed during scan matching, leading to only a small change in the final SLAM localization.

This behavior is consistent with the role of scan matching in refining pose estimates in LiDAR-based SLAM systems [3,9]. Improved odometry provides a better starting estimate, but the final localization accuracy may depend on how effectively the SLAM algorithm performs scan matching and pose optimization.

### 5.2. Differences Between SLAM Algorithms

The three SLAM algorithms responded differently to the improved odometry. GMapping showed small translational changes between odometry sources, while rotational performance improved for a majority of the evaluated trajectories. Karto SLAM and SLAM Toolbox had a majority of their trajectory conditions result in worse performance due to the improved odometry source.

The evaluated SLAM algorithms differ in their internal use of odometry, scan matching, loop closure, and pose optimization. GMapping uses a Rao-Blackwellized particle filter with scan matching, whereas Karto SLAM and SLAM Toolbox use graph-based formulations with scan matching and optimization [9,11,14]. These architectural differences may contribute to the different responses observed across algorithms. However, because the present experiments did not independently vary scan matcher type, update thresholds, optimization settings, or loop closure behavior, the contribution of each mechanism cannot be isolated. These results should therefore be interpreted as algorithm-dependent trends for the evaluated dataset and configurations rather than as evidence that a particular scan-matching architecture caused the observed differences.

### 5.3. Influence of Trajectory Characteristics

The effectiveness of EKF-based sensor fusion varied with the characteristics of the evaluated trajectories. Prior to SLAM, sensor fusion reduced translational APE RMSE by 61.5%, 61.0%, 74.6%, and 71.0% for Trajectories A–D, respectively. Rotational APE RMSE followed a similar trend with 65.1%, 70.3%, 77.2%, and 74.6%. Trajectory C exhibited the greatest relative improvement for both translational and rotational errors. Although these specific motion characteristics were not independently isolated, the variation among segments indicates that the magnitude of the EKF improvement varied with the evaluated trajectory. After SLAM execution, trajectory-dependent behavior was more evident in rotational localization. For SLAM Toolbox, the filtered odometry reduced rotational APE RMSE by 36.9% for Trajectory B and 24.0% for Trajectory D, while increasing rotational APE RMSE by 22.6% for Trajectory A and 29.7% for Trajectory C. GMapping similarly shifted from an increase in rotational error for A to reductions of 14.8–18.5% for the remaining trajectories. Because Trajectories A–C are subsets of Trajectory D, the effects of trajectory length, angular velocity, and environmental geometry cannot be separated from the present results. Consequently, the observed differences should be interpreted as evidence of trajectory-dependent behavior rather than a causal relationship between trajectory characteristics and SLAM performance.

## 6. Conclusions

This study assessed the influence of odometry quality on SLAM localization performance. Multiple SLAM algorithms were evaluated using two localization pipelines: raw wheel odometry and odometry fused with IMU measurements through an Extended Kalman Filter (EKF). Localization performance was evaluated both before and after SLAM to isolate the performance improvements provided by sensor fusion from those introduced by the SLAM algorithm.

Prior to SLAM, EKF-based sensor fusion reduced localization error by approximately 60–75% translationally and 65–77% rotationally across all evaluated trajectories compared to raw wheel odometry. However, these improvements did not translate into corresponding reductions in post-SLAM localization error. Post-SLAM differences in translational APE RMSE were substantially smaller and varied according to the evaluated algorithm and trajectory, while rotational performance exhibited larger and less consistent changes. These results indicate that the evaluated SLAM algorithms are able to compensate for a substantial portion of the odometric error, reducing the influence of sensor fusion on the final localization accuracy.

By independently evaluating odometry and end-to-end SLAM localization, this study provides insight into how improvements in odometry quality influence overall localization performance. These findings demonstrate that improvements in odometry quality do not always translate into proportional improvements in end-to-end SLAM performance, emphasizing that the effectiveness of sensor fusion ultimately depends on how individual SLAM algorithms utilize odometric information during localization.

Because the evaluation was conducted using a single recorded traversal, the findings should be interpreted within the evaluated dataset, algorithms, and configurations. The repeated executions characterize SLAM run-to-run variability but do not constitute independent environmental or traversal samples.

Future work will evaluate independently collected trajectories designed to systematically vary characteristics such as trajectory length, turning frequency, angular velocity, and environmental geometry.

## Figures and Tables

**Table 1:** Key default configuration parameters for the evaluated SLAM algorithms.

Parameter	GMapping	Karto	SLAM Toolbox
Map Resolution (m/cell)	0.05	0.05	0.05
Linear Update Threshold (m)	0.5	0.2	0.5
Angular Update Threshold (rad)	0.5	0.1	0.5
Scan Matching Setting	Enabled	Enabled	Enabled
Loop Closure	-	Enabled	Enabled
Particles	30	-	-
Other	Package Default	Package Default	Package Default

**Table 2:** Localization error metrics for raw and EKF-filtered odometry relative to the ground truth trajectory.

Trajectory	Localization	Trans. APE RMSE (m)	Trans. APE Mean (m)	Trans. APE Max (m)	Rot. APE RMSE (°)	Rot. APE Mean (°)	Rot. APE Max (°)
A	Raw Odometry	1.5519	1.1987	5.5075	13.7612	11.6892	32.4490
A	EKF	0.5970	0.4736	1.7555	4.8012	3.9891	9.9658
B	Raw Odometry	1.7759	1.3454	4.2049	21.9908	18.9552	43.1386
B	EKF	0.6922	0.5730	2.1225	6.5227	5.4124	11.8877
C	Raw Odometry	1.9777	1.6324	5.9922	14.9173	12.7804	29.8917
C	EKF	0.5027	0.4467	1.3463	3.4058	2.9905	6.3294
D	Raw Odometry	9.5720	7.6384	27.3603	49.3980	42.8552	101.6395
D	EKF	2.7716	2.2715	7.7738	12.5644	10.9136	24.6316

**Table 3:** Post-SLAM localization performance using the raw and EKF-filtered odometry across the evaluated SLAM algorithms and trajectories. Negative Δ APE RMSE percentages indicate increased localization error relative to raw odometry sourced SLAM while positive values indicate reduced error. Values represent mean APE RMSE ± sample standard deviation across five executions.

Algorithm	Trajectory	Raw Odometry Trans. APE RMSE (m)	EKF Trans. APE RMSE (m)	Δ Trans. APE RMSE (%)	Raw Odometry Rot. APE RMSE (°)	EKF Rot. APE RMSE (°)	Δ Rot. APE RMSE (%)
GMapping	A	0.3474 ± 0.0105	0.3466 ± 0.0107	0.2%	0.8670 ± 0.1418	0.9983 ± 0.0597	−15.2%
GMapping	B	0.2877 ± 0.0139	0.2928 ± 0.0148	−1.8%	0.7494 ± 0.0711	0.6386 ± 0.0886	14.8%
GMapping	C	0.3503 ± 0.0048	0.3394 ± 0.0052	3.1%	0.7145 ± 0.0645	0.5821 ± 0.0289	18.5%
GMapping	D	0.3749 ± 0.0242	0.3671 ± 0.0143	2.1%	1.0863 ± 0.2125	0.8868 ± 0.1085	18.4%
Karto	A	0.2399 ± 0.0010	0.2578 ± 0.0136	−7.5%	0.3864 ± 0.0074	0.7737 ± 0.0222	−100.2%
Karto	B	0.3008 ± 0.0016	0.3083 ± 0.0021	−2.5%	0.4625 ± 0.0379	0.4178 ± 0.0268	9.7%
Karto	C	0.3826 ± 0.0082	0.4388 ± 0.0285	−14.7%	0.6547 ± 0.0256	0.7092 ± 0.0450	−8.3%
Karto	D	0.2912 ± 0.0152	0.3041 ± 0.0262	−4.4%	0.4951 ± 0.0390	0.6367 ± 0.0267	−28.6%
Toolbox	A	0.2748 ± 0.0001	0.2755 ± 0.0020	−0.2%	0.5925 ± 0.0044	0.7264 ± 0.0087	−22.6%
Toolbox	B	0.2710 ± 0.0025	0.2780 ± 0.0004	−2.6%	0.7060 ± 0.0510	0.4456 ± 0.0098	36.9%
Toolbox	C	0.3572 ± 0.0014	0.3663 ± 0.0002	−2.5%	0.5509 ± 0.0222	0.7144 ± 0.0073	−29.7%
Toolbox	D	0.3636 ± 0.0198	0.3578 ± 0.0652	1.6%	0.9305 ± 0.0317	0.7068 ± 0.0086	24.0%

**Table 4:** Welch’s *t*-test comparison of the raw odometry and EKF-filtered post-SLAM APE RMSE across five repeated executions per condition. “EKF lower” indicates that the EKF condition produced a lower mean APE RMSE than the raw-odometry condition, while “EKF higher” indicates a higher mean APE RMSE. Results with *p* ≥ 0.05 were treated as not statistically distinguishable from run-to-run variability. Statistical results are interpreted as within-dataset evidence rather than population-level generalization.

Algorithm	Trajectory	Trans. *p*-Value	Trans. Result	Rot. *p*-Value	Rot. Result
GMapping	A	0.905	Not Distinguishable	0.111	Not Distinguishable
GMapping	B	0.593	Not Distinguishable	0.062	Not Distinguishable
GMapping	C	0.009	EKF Lower	0.007	EKF Lower
GMapping	D	0.555	Not Distinguishable	0.111	Not Distinguishable
Karto	A	0.042	EKF Higher	<0.001	EKF Higher
Karto	B	<0.001	EKF Higher	0.067	Not Distinguishable
Karto	C	0.01	EKF Higher	0.054	Not Distinguishable
Karto	D	0.375	Not Distinguishable	<0.001	EKF Higher
Toolbox	A	0.497	Not Distinguishable	<0.001	EKF Higher
Toolbox	B	0.003	EKF Higher	<0.001	EKF Lower
Toolbox	C	<0.001	EKF Higher	<0.001	EKF Higher
Toolbox	D	0.856	Not Distinguishable	<0.001	EKF Lower

## Data Availability

The data presented in this study are available on request from the corresponding author due to restrictions related to data sharing and access.

## Appendix A

### Appendix A.1

The configuration vector and covariance matrix order follows the standard robot_localization state ordering: *x*, *y*, *z*, *φ*, *θ*, *ψ*, $\dot{x}$, $\dot{y}$, $\dot{z}$, $\dot{\theta }$, $\dot{\varphi }$, $\dot{\psi }$, $\ddot{x}$, $\ddot{y}$, $\ddot{z}$. The process noise covariance and initial estimate covariance matrices were diagonal; all off-diagonal terms were zero.

**Table A1:** robot_localization EKF configuration used for the EKF-filtered odometry condition.

Parameter	Value
ROS Package	robot_localization
Filter Node	ekf_localization_node
Filter Frequency	100 Hz
Sensor Timeout	0.5 s
Simulation Time	use_sim_time: true
Two-Dimensional Mode	two_d_mode: true
Map Frame	map
Odometry Frame	odom
Base Frame	base_link
World Frame	odom
TF Publication	publish_tf: true
Diagnostics	print_diagnostics: true
Odometry Input Topic	/odom_inflated
Odometry Measurement Covariance	Assigned in the incoming/odom_inflated message prior to EKF fusion; not specified in the robot_localization YAML file. The longitudinal velocity covariance was set to 0.01.
Odometry Differential Mode	odom0_differential: false
Odometry Fused Variable	Longitudinal velocity, ($\dot{x}$)
Odometry Configuration Vector	[false, false, false, false, false, false, true, false, false, false, false, false, false, false, false]
IMU Input Topic	/torso_lift_imu/data
IMU Differential Mode	imu0_differential: false
IMU Relative Mode	imu0_relative: false
IMU Gravitational Acceleration Removal	imu0_remove_gravitational_acceleration: true
IMU Fused Variable	Yaw angular velocity, ($\dot{\psi }$)
IMU Configuration Vector	[false, false, false, false, false, false, false, false, false, false, false, true, false, false, false]
Configuration Vector Order	(*x*, *y*, *z*, *φ*, *θ*, *ψ*, $\dot{x},$ $\dot{y},$ $\dot{z},$ $\dot{\varphi },$ $\dot{\theta },$ $\dot{\psi },$ $\ddot{x},$ $\ddot{y},$ $\ddot{z}$)
Process Noise Covariance Diagonal	[0.05, 0.05, 0.06, 0.03, 0.03, 0.06, 0.025, 0.025, 0.04, 0.01, 0.01, 0.02, 0.01, 0.01, 0.015]
Initial Estimate Covariance Diagonal	[1 × 10^−3^, 1 × 10^−3^, 1 × 10^−3^, 1 × 10^−3^, 1 × 10^−3^, 1 × 10^−3^, 1 × 10^−3^, 1 × 10^−3^, 1 × 10^−3^, 1 × 10^−3^, 1 × 10^−3^, 1 × 10^−3^, 1 × 10^−3^, 1 × 10^−3^, 1 × 10^−3^]
Process Noise and Initial Estimate Covariance Off-Diagonal Terms	0
Other Parameters	Package default values

### Appendix A.2

**Table A2:** TF publication validation comparing original EKF results generated using robot_localization TF publication with validation results generated using the custom odometry-to-TF broadcaster. Values are calculated as validation mean minus the original EKF mean. Negative values indicate that the validation pipeline produced a lower RMSE than the original EKF pipeline. The validation used three repeated executions for each SLAM algorithm and Trajectories A–C.

Algorithm	Trajectory	Δ Trans. RMSE (m)	Δ Trans. RMSE (%)	Δ Rot. RMSE (°)	Δ Rot. RMSE (%)
GMapping	A	−0.0004	−0.12	−0.0700	−7.01
GMapping	B	0.0040	1.38	−0.0283	−4.43
GMapping	C	0.0047	1.38	0.0987	16.96
Karto	A	−0.0065	−2.52	−0.0127	−1.64
Karto	B	0.0004	0.13	−0.0042	−1.01
Karto	C	0.0081	1.86	0.0189	2.67
Toolbox	A	−0.0020	−0.72	0.0099	1.36
Toolbox	B	0.0004	0.14	−0.0021	−0.48
Toolbox	C	0.0001	0.02	−0.0038	−0.54

## References

[1] Kam M., Zhu X., Kalata P. (1997). Sensor Fusion for Mobile Robot Navigation. Proc. IEEE.

[2] Alatise M.B., Hancke G.P. (2020). A Review on Challenges of Autonomous Mobile Robot and Sensor Fusion Methods. IEEE Access.

[3] Ran Y., Xu X., Tan Z., Luo M. (2025). A Review of 2D Lidar SLAM Research. Remote Sens..

[4] Kim Y., Bang H., Govaers F. (2019). Introduction to Kalman Filter and Its Applications. Introduction and Implementations of the Kalman Filter.

[5] Nguyen Q.H., Johnson P., Latham D. (2023). Performance Evaluation of ROS-Based SLAM Algorithms for Handheld Indoor Mapping and Tracking Systems. IEEE Sens. J..

[6] Trejos K., Rincón L., Bolaños M., Fallas J., Marín L. (2022). 2D SLAM Algorithms Characterization, Calibration, and Comparison Considering Pose Error, Map Accuracy as Well as CPU and Memory Usage. Sensors.

[7] Wang H., Shi Z., Li C., Wang F., Zhang S., Zhang Y. (2023). Comparison and Implementation of ROS-Based SLAM and Path Planning Methods. Artificial Intelligence Logic and Applications.

[8] Zhao J., Liu S., Li J. (2022). Research and Implementation of Autonomous Navigation for Mobile Robots Based on SLAM Algorithm under ROS. Sensors.

[9] Olson E.B. Real-Time Correlative Scan Matching. Proceedings of the 2009 IEEE International Conference on Robotics and Automation.

[10] Moore T., Stouch D., Menegatti E., Michael N., Berns K., Yamaguchi H. (2016). A Generalized Extended Kalman Filter Implementation for the Robot Operating System. Intelligent Autonomous Systems 13.

[11] Grisetti G., Stachniss C., Burgard W. (2007). Improved Techniques for Grid Mapping With Rao-Blackwellized Particle Filters. IEEE Trans. Robot..

[12] Konolige K., Grisetti G., Kümmerle R., Burgard W., Limketkai B., Vincent R. Efficient Sparse Pose Adjustment for 2D Mapping. Proceedings of the 2010 IEEE/RSJ International Conference on Intelligent Robots and Systems.

[13] Quigley M., Gerkey B., Conley K., Faust J., Foote T., Leibs J., Berger E., Wheeler R., Ng A. ROS: An Open-Source Robot Operating System. Proceedings of the ICRA Workshop on Open Source Software.

[14] Macenski S., Jambrecic I. (2021). SLAM Toolbox: SLAM for the Dynamic World. JOSS.

[15] Lynch K.M., Park F.C. (2017). Modern Robotics: Mechanics, Planning, and Control.

[16] Sturm J., Engelhard N., Endres F., Burgard W., Cremers D. A Benchmark for the Evaluation of RGB-D SLAM Systems. Proceedings of the 2012 IEEE/RSJ International Conference on Intelligent Robots and Systems.

[17] Fallon M., Johannsson H., Kaess M., Leonard J.J. (2013). The MIT Stata Center Dataset. Int. J. Robot. Res..

[18] Grupp M. Evo: Python Package for the Evaluation of Odometry and SLAM, v1.37.0. https://github.com/MichaelGrupp/evo.

